# pH-driven solubilization and isoelectric precipitation of proteins from the brown seaweed *Saccharina latissima*—effects of osmotic shock, water volume and temperature

**DOI:** 10.1007/s10811-016-0957-6

**Published:** 2016-09-23

**Authors:** Jenny Veide Vilg, Ingrid Undeland

**Affiliations:** Food and Nutrition Science, Biology and Biological Engineering, Chalmers University of Technology, 41296 Gothenburg, Sweden

**Keywords:** *Saccharina latissima*, Phaeophyceae, Protein, Seaweed, Solubility, Precipitation, pH

## Abstract

In the light of the global search for novel and sustainable protein sources, macroalgal proteins are becoming an attractive target. To date, mainly red and green macroalgae have been investigated in this respect, whereas the brown species are less studied, possibly because of the lower content of protein. In a biorefinery context, however, the protein content of brown macroalgae can still be economically interesting due to fast growth and the possibility to co-extract other compounds, such as alginates. The aim of this study was to develop a simple, scalable pH shift-based protein isolation technique applicable on wet *Saccharina latissima* biomass. Factors investigated were extraction volume, temperature, protein solubilization pH, osmoshock pretreatment and protein precipitation pH. Maximum protein solubility was obtained at pH 12, where 34 % of the total protein content could be extracted with 5.56 volumes of extraction solution (20 volumes on dry weight (dw) basis). Osmoshocking significantly increased the yield, and 20, 40 and 60 volumes of water (dw basis) gave 45.1, 46.8 and 59.5 % yield, respectively. The temperature during osmoshocking did not significantly affect the extraction yield, and extended time (16 vs. 1 or 2 h) reduced protein yield. Precipitation of solubilized proteins was possible below pH 4; the highest precipitation yield, 34.5 %, was obtained at pH 2. After combined alkaline extraction and acid precipitation, 16.01 % of the *Saccharina* proteins were recovered, which can be considered acceptable in comparison to other studies on algae but leaves some room for improvement when compared to protein extraction from, for instance, soy.

## Background

The growing global human population, with increasing demands for nutrient-rich foods, constitutes a great challenge when it comes to adequate protein supply, both for human consumption and for feed that can be converted into animal source protein (Wu et al. 2014). Novel sources of protein will make an important contribution to the world’s supply in the future, but we need to consider not only the productivity, but also sustainability concerns to avoid depletion of water and land reserves (Boland et al. 2013). The interest in marine seaweeds as a substrate for biorefining has increased, with products such as bioethanol (Daroch et al. 2013) biogas (Hughes et al. 2012), polymers (Bixler and Porse 2011), antioxidants (Jónsdóttir et al. 2016) and proteins (e.g. Harnedy and FitzGerald 2013) in focus. An important advantage with using seaweed biomass as a substrate is that their cultivation does not require arable land, nor irrigation, fertilization or pesticides, which increases the potential for sustainable production. There is a varying content of protein in macroalgae, depending on species, and the brown kelps, which are most commonly described in biorefinery contexts, generally contain high concentrations of carbohydrates but have a lower protein content (Fleurence 1999). However, considering their high productivity (Kraan 2013), the yield per cultivation area could still be competitive as compared to other species.

Macroalgal proteins have been reported to have an amino acid profile suitable for human consumption (Maehre et al. 2014; Marinho et al. 2015), and eating seaweeds is a part of the cuisine in several countries. The bioavailability of the algal proteins has been discussed; for instance, the soluble fibres of seaweed have been shown to inhibit the digestibility of protein (Horie et al. 1995)_._ This gives a good reason for extracting the protein fraction from the rest of the algal matrix, before using it for consumption. Other positive effects of extraction are that the end product becomes more nutrient dense and that other fractions can be separated in parallel, as in a biorefinery concept, which increases the economic value (Hou et al. 2015). There are few reports on methods for extraction of seaweed proteins, especially from the brown seaweeds. Jordan and Vilter (1991) showed how the occurrence of phenolic compounds, pigments and large amounts of polyanionic cell wall mucilages, mainly consisting of alginates, renders protein extraction from this class of seaweed difficult.

The fact that proteins in water obtain net positive or negative charges when adjusted to extremely acid or alkaline conditions, respectively, can be the basis for their isolation. This is since the strong repulsions caused by like charges favour protein solubilization, whereupon non-soluble matter can be removed, e.g. by centrifugation. Alkaline solubilization followed by isoelectric protein precipitation is applied, for example, in the isolation of soy protein (Rickert et al. 2004) and wheat protein (Liu et al. 2013). Also, in the early 2000s, acid or alkaline solubilization, followed by removal of non-solubles and isoelectric precipitation, was introduced as a principle to isolate boneless and nearly lipid-free proteins from complex raw materials like whole fish or fish by-products (e.g. Hultin and Kelleher 1999, 2000, 2001; Hultin et al. 2000; Undeland et al. 2002). Performing the whole procedure under cold conditions allowed the fish proteins to retain their technical functionality, including their capacity to form a gel. This procedure, which often goes under the name ‘pH shift method,’ has also been applied, e.g. to shellfish (Vareltzis and Undeland 2012) and recently to microalgae (Cavonius et al. 2015, 2016).

Little has been done on pH shift-like processing of seaweed; the few papers that exist are on red seaweeds, *Palmaria palmata* (Harnedy and FitzGerald 2013, 2015) and *Kappaphycus alvarezii* (Doty) (Kumar et al. 2014), various species of green seaweed (Fleurence et al. 1995; Kandasamy et al. 2012), or subtropical brown seaweeds (three *Sargassum* species) (Wong and Cheung 2001). Kandasamy et al. (2012), Kumar et al. (2014) and Wong and Cheung (2001), however, precipitated the proteins with ammonium sulphate, which then required a dialysis step. Fleurence et al. (1995) and Harnedy and FitzGerald (2013) focused only on the first part of the process, the alkaline solubilization, with and without osmotic shock, high shear treatments or addition of polysaccaridase enzymes (e.g. Celluclast 1.5 L and Shearzyme 500 L), to break down the cellular structures. The authors, however, concluded that since high enzyme/substrate ratios were required, applying these polysaccharidases may not be feasible for the extraction of intact *P. palmata* proteins. In some of the mentioned studies (e.g. Kumar et al. 2014), the water-soluble proteins have been removed in a first step, at the end of the osmotic shock treatment, and have then been combined with the alkali-soluble ones prior to precipitation. Several studies have also applied reducing agents along with the alkaline solubilization (2-mercaptoethanol or *N*-acetyl-l-cysteine (NAC)) (Wong and Cheung 2001; Kandasamy et al. 2012; Harnedy and FitzGerald 2013; Kumar et al. 2014), e.g. to break S–S bonds. The mentioned studies have also used all dried algae biomass.

To make a protein recovery process economically feasible and easy to apply in industry, a simplistic approach with a minimum amount of low energy steps would be beneficial. Bearing this goal in mind, the aim of the present study was to develop basic settings for a relatively simple pH-driven protein extraction protocol to apply directly on wet brown seaweed biomass. Using *Saccharina latissima* as a focus species, we wished to evaluate the optimal pH for protein solubilization, the impact from the water to biomass ratio used and the inclusion of an osmotic shock step varying in time and temperature. Brief investigation of pH-driven precipitation of the extracted proteins was also to be carried out, to develop a full pH shift process protocol.

## Methods

### Materials and general biomass preparation

The algal biomass used for all experiments in this study was from wild specimens of *Saccharina latissima*, collected by diving in the archipelago of the Swedish west coast in November 2013. Here, the algae grow in a mildly exposed environment, with a tidal amplitude of 10 cm, temperatures between 6 and 20 °C and a salinity around 3 psu. The fresh biomass was minced using a meat grinder (Bankeryds Maskin AB, Sweden), with three layers of hole plates with openings of 450, 5 and 2 mm, respectively, and stored at −20 °C in ziplock bags until use. Before the experiments, the algal biomass was weighed in frozen state and distilled water added to the *w*/*w* ratio needed for the specific experiment. All experimental biomass was kept on ice during the whole process unless stated otherwise.

### Total protein analysis

For the analysis of total protein concentration of algal biomass or residual pellets, we evaluated six different extraction methods before selecting the one with results corresponding best to a reference Kjeldahl analysis. The material was freeze-dried and finely ground in a mortar. Around 50 mg of the powder was added to 1 mL of extraction liquid and treated as follows: (1) Milli-Q water, 1 h at room temperature; (2) Milli-Q water, pH 11, 1 h at room temperature; (3) Milli-Q water, 1 h at 80 °C; (4) 2 % SDS, 1 mM dithiothreitol (DTT), 100 °C, 3 × 5 min with mixing by vortex in between; (5) 2 % SDS, 1 mM DTT, pH 11, 100 °C, 3 × 5 min with mixing by vortex in between; and (6) 2 × 500 μL 2 % SDS, 1 mM DTT, pH 11, 100 °C, 3 × 5 min with mixing by vortex in between (the 2 × 500-μL extracts were pooled). The extracts were separated from the solid residuals by centrifugation at 4 °C, 14,000×*g* for 20 min in a microcentrifuge (Eppendorf 5417R). From the results of the subsequent Lowry analysis, we chose treatment no. 4 as the extraction method for total protein analysis. For protein analyses of final precipitates from the pH shift experiment, the entire pellets were dissolved in 500 μL extraction buffer (i.e. treatment no. 4) and boiled for 3 × 5 min to denature the proteins before analysis.

The above samples, along with supernatants from the described extraction sequences (initial seaweed extracts, solubilized proteins and precipitated proteins), were analyzed using the DC Protein assay (Bio-Rad), which is based on the Lowry assay (Lowry et al. 1951), in microwell format. Bovine serum albumin was used as a standard, and the reliability of the method for algal biomass was confirmed by comparison with Kjeldahl total protein analysis (Kjeldahl, 1883), using a conversion factor of 5.6 (Bogolitsyn et al. 2014).

### Determination of optimal pH for solubilizing the algal proteins

To determine the protein solubility at different pH values, 20 g algal biomass was mixed with water to a ratio of 1:4 (wet weight) and homogenized with a polytron (UltraTurrax T18 basic) at speed 4 for 2 min. pH was adjusted to a range of values between 2 and 13 by addition of hydrochloric acid (HCl) or sodium hydroxide (NaOH) (0.1 or 1 M), and the slurry was left to incubate for 20 min with stirring on ice. Samples of 1.5 mL were taken in triplicate, and the supernatant was separated by centrifugation at 8000×*g* for 10 min (Thermo Fisher Scientific, Heraeus Fresco 17, Germany). The concentration of soluble protein was determined by the Lowry method as described previously. Solubility was expressed as protein concentration in the supernatant divided by the total protein concentration in the slurry × 100. The point where maximum solubility was obtained (pH 12, see “Results” section) was used in further trials described.

### Development of a protein extraction sequence

A basic extraction sequence was set up with a wet algal biomass to water ratio of 1:5.56, corresponding to 1:20 dry weight biomass/water. Algal biomass (13.22 g) per replicate was suspended in water to a total mass of 50 g in 200-mL beakers. Thereafter, the suspension was homogenized using a polytron (UltraTurrax T18 basic) for 2 min at speed 4.

The homogenized biomass was first osmoshocked, i.e. incubated for 2 h at 4 °C with shaking at 150 rpm in the Milli-Q water in which it was homogenized. The slurry was then centrifuged at 5525×*g* for 30 min, and the supernatant was separated for analysis of water-soluble proteins (see “Total protein analysis” section). The pellet was resuspended in water to a total of 50 g, adjusting pH to 12 by adding 1 M NaOH. After incubation for 60 min at pH 12, 4 °C and shaking at 150 rpm, the solid fraction was again separated by centrifugation at 5525×*g* for 30 min. The content of alkaline-soluble proteins in the supernatant was analyzed as described previously. The pellet was, when stated, freeze-dried and ground in a mortar before extraction and analysis of total remaining protein.

**Fig. 1:**
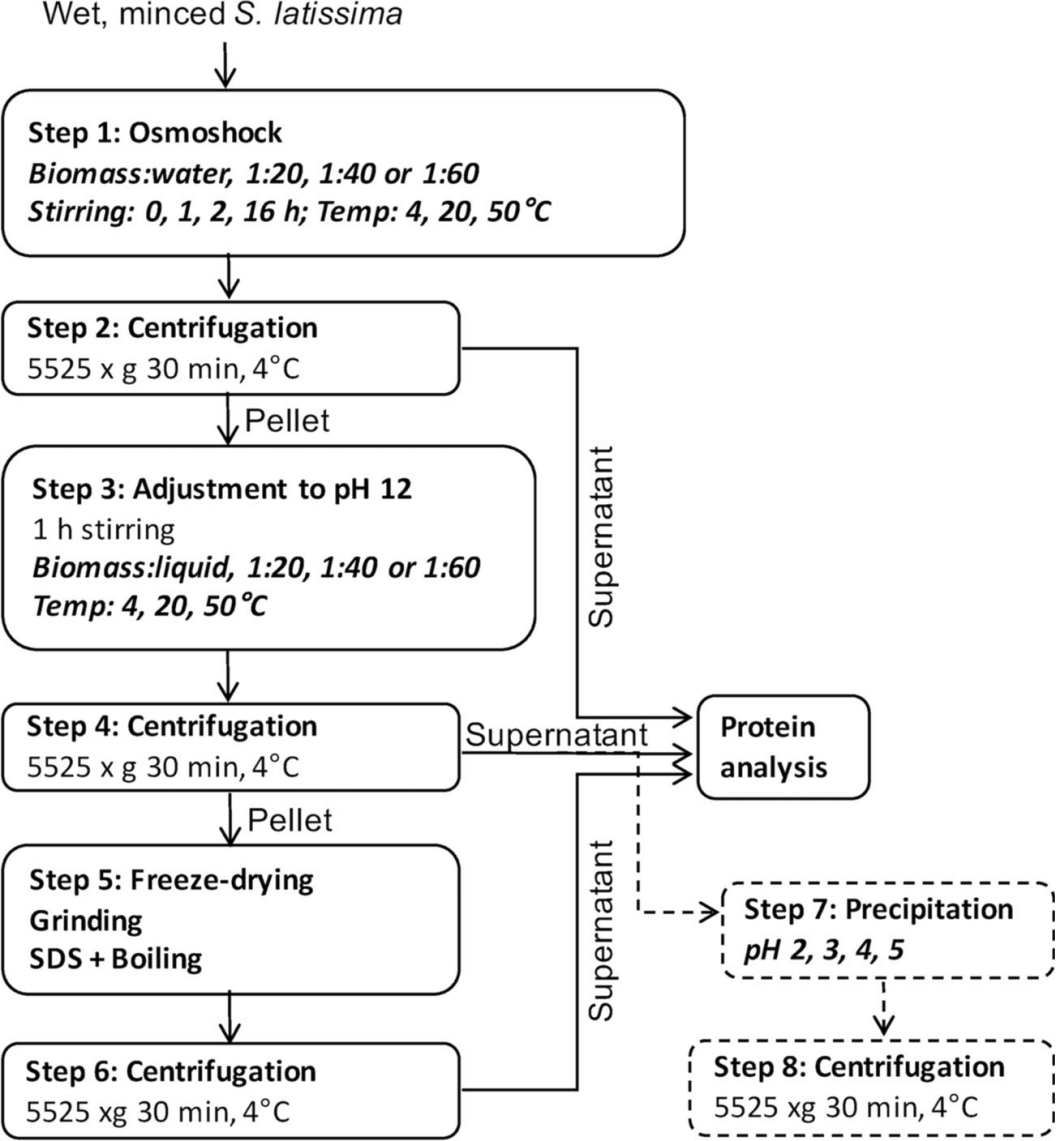
Protein extraction sequence. The scheme describes the solubilization procedure and gives an overview of the variations in biomass/water ratio, length of osmoshock and temperature. The part shown in *dashed lines* represents the subsequent precipitation sequence

To optimize the extraction sequence, experiments were carried out with variation in (i) temperature (4, 20 and 50 °C), (ii) ratio between amount of biomass and volume of water in the osmoshock step and alkaline extraction (1:20, 1:40, 1:60, as calculated on a biomass dry weight (dw) basis) or (iii) length of osmotic shock (0, 1, 2, 16 h. The abovedescribed protocol was used as a basis but with specific adjustments for the selected parameters (see Fig. 1 for overview).

### Precipitation of alkali-extracted proteins to develop a pH shift process

For the precipitation experiments, which were designed to develop a pH shift-based protein extraction method, protein extract was prepared as follows: frozen algal biomass was added to distilled water at a ratio of 1:60 (dry weight, corresponding to 1:11 wet weight algal biomass/water) and homogenized with a polytron as described previously. An incubation step of 1 h at 20 °C at the native pH (pH 7.2) then followed, for osmotic shock, whereafter the pH was directly adjusted to pH 12 with 1 M NaOH, without prior separation of the water extract. The homogenate was then incubated at pH 12 at room temperature for 1 h, after which the supernatant was separated by centrifugation at 5525×*g* (4 °C) for 30 min.

Seven millilitre of the supernatant was aliquoted to five 15-mL Falcon tubes, and the pH was adjusted to values between 2 and 5 with 1 M HCl in four of the tubes, whereas one was left as control (pH 12). The precipitated proteins were subsequently separated by centrifugation at 5525×*g* (4 °C) for 30 min, and both supernatant and precipitate were analyzed for protein concentration as described. The experiment was performed in triplicate.

### Salinity analysis of algal biomass

Eighteen millilitre of Milli-Q water was added to 2 g of algal biomass in a 50-mL tube and homogenized with a polytron at speed 5 for 30 s. The conductivity of the homogenate was determined with a conductivity meter (MeterLab PHM210, Radiometer analytical S.A., France). Three replicates were prepared for each sample. Conductivity was converted to NaCl equivalent through a standard curve constructed with NaCl solution (0–500 mM).

### Statistics and expression of data

All experiments were made in triplicates if not described differently. The algae-water slurries were not homogenous and needed extensive mixing before sampling to obtain repeatable results. Depending on experiment, the data is expressed as milligram protein per gram algal biomass, protein solubility percentage:Solubility (%) = C_soluble proteins in supernatant_ / C_total protein in homogenate_ × 100or protein yield percentage:Yield (%) = M_extracted protein in supernatant_ / M_total protein in homogenate_ × 100

To statistically verify the differences between means, we used SPSS (IBM, USA) software to calculate the statistical significance with independent sample *t* test. The equality of variances between groups was calculated with Levene’s test.

## Results

### Method for total protein determination

To find a simple, yet reliable analysis method for determination of total protein concentration in algal samples and extracts/pellets thereof, we compared different extraction procedures, in combination with the Lowry quantification principle. The latter method has been shown previously to be more suitable for protein analysis of algal biomass than the Bradford method (Barbarino and Lourenco 2005).

**Fig. 2:**
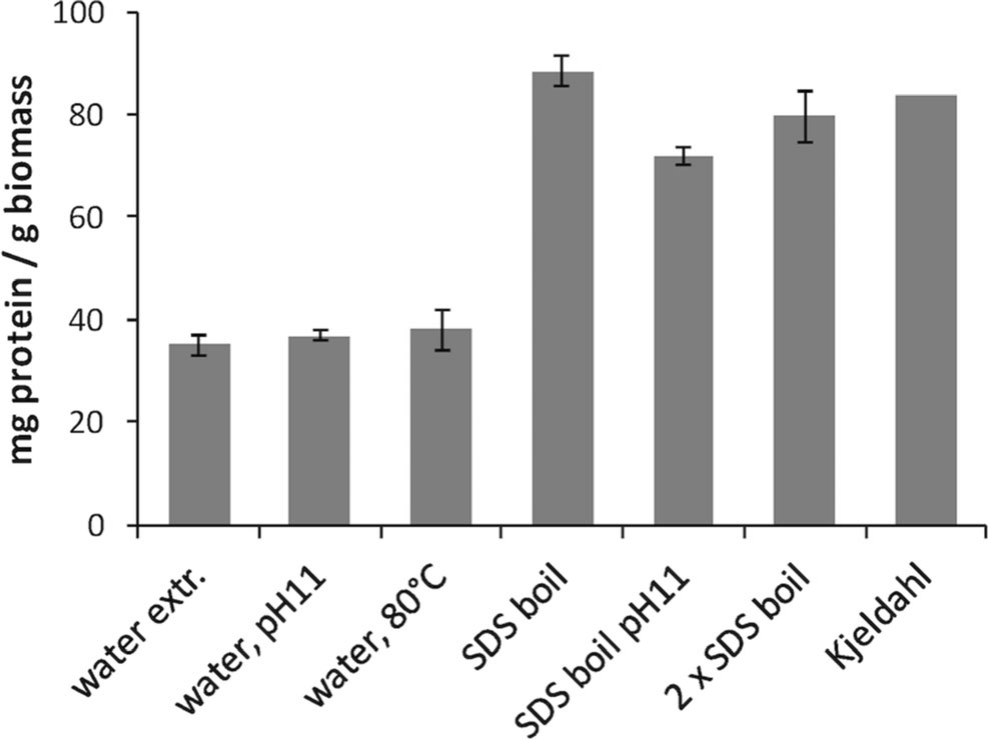
Assessment of extraction methods for total protein analysis. Different methods for extracting total protein for subsequent Lowry analysis were compared. Freeze-dried and finely ground algal biomass was subjected to extraction by (1) water, room temperature, 1 h; (2) water, pH 11, room temperature, 1 h; (3) water, 80 °C, 1 h; (4) 2 % SDS + 1 mM dithiothreitol (DTT), 100 °C, 3 × 5 min; (5) 2 % SDS + 1 mM DTT, pH 11, 100 °C, 3 × 5 min; and (6) 2 % SDS + 1 mM DTT, pH 11, 100 °C, 3 × 5 min in two steps. The same biomass was analyzed with (7) Kjeldahl method, for comparison

Water extraction of samples at a high pH (>pH 11) or at high temperature (80 °C) gave a low protein yield, as compared to the 84 mg protein g^−1^ biomass that was revealed by the reference total nitrogen analysis by Kjeldahl (Fig. 2). A harsher method of boiling the samples at their native pH in 2 % SDS and 1 mM DTT, on the other hand, yielded 88 mg g^−1^ biomass, which is comparable to the value calculated from Kjeldahl-derived total nitrogen values. Increasing the pH of the extraction solution to pH 11 did not further improve the extraction. Since the boiling in SDS gave a value that was comparable to the result from the analysis according to the Kjeldahl method, this method was used for protein determination throughout the experiments.

### Optimization of pH for algal protein solubilization in water

**Fig. 3:**
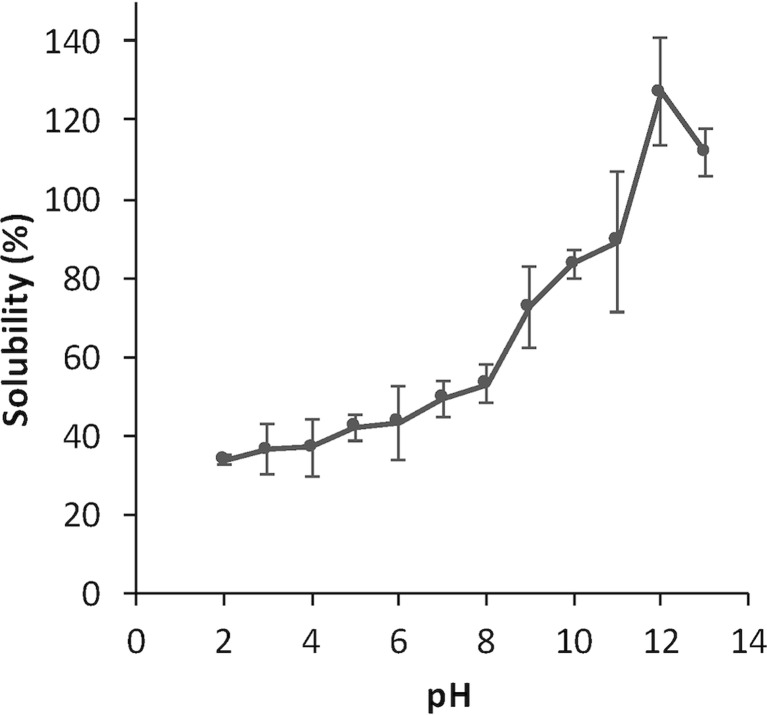
pH-dependent solubility. Slurries with homogenized algal biomass and water in a ratio of 1:4 were incubated at different pH values, before separating liquid and solid material by centrifugation. Total protein content and solubilized proteins were quantified, and the solubility is expressed as a percentage as calculated from the extract concentration divided by the total protein concentration of the original slurry. All experiments are performed in triplicates, and the *error bars* represent standard deviations

To map the protein solubility in water at different pH values, for accurate design of the extraction procedure, algal biomass was homogenized in water at a ratio of 1:4 (*w*/*w*), and pH was adjusted to values between 2 and 12 as indicated in Fig. 3. Our results showed that the solubility peaked at pH 12, with over 100 % solubility. This slight overestimation could be due to sampling differences, since the algal slurry was not absolutely homogenous. The solubility was decreasing with decreasing pH values, eventually plateauing at pH 2–3, where the solubility was only about 30 %. The pattern of protein solubility of the macroalgal biomass differs from those reported, for example, for nuts (Ramos and Bora 2004), whey (Mulcahy et al. 2016) and fish (Undeland et al. 2003), the curves of which were U-shaped with a clear dip in solubility around pH 4–6. It is, however, in accordance with other studies of marine algal proteins, with the same slope-shaped solubility curves. For instance, the solubility of *Nannochloropsis oculata* protein has been shown to remain low from pH 3 down to 1 (Cavonius et al. 2015). The isoelectric point (pI) generally appears to be lower in algae proteins than in other biomasses; values of pH 3.5 for *Scenedesmus acutus* (Venkataraman and Shivashankar 1979), pH 3.0 for *Spirulina platensis* (Devi et al. 1981), and pH 4 for *Tetraselmis* sp*.* (Schwenzfeier et al. 2011) have been reported, but the lack of increase in solubility at lower pH seems to be unique for the marine species. This is probably an effect of the salt concentrations in the experiments, the interaction of anions with positively charged groups of proteins at low pH being a well-known mechanism pushing the pI downwards (Belitz et al. 2009).

Since the pH-dependent protein solubility is known to be affected by the ionic strength, we analyzed the salinity of the algal biomass. The conductivity, as expressed in NaCl equivalents, was as high as 525 ± 8.28 mM in the wet biomass, which means 80 mM in a 1:5.56 water homogenate. This reflects the enrichment of minerals (e.g. Na, K, Mg, Ca) in brown seaweed (Ruperez 2002) and their counter anions, and could be the cause of the low solubility at low pH and lack of distinct pI in our study. It has, in fact, been shown previously, with *Tetraselmis* sp., that the ionic strength could affect the solubility. In that study, it was shown not only that higher salt concentrations lowered the solubility at low pH values but also that they cause a lack of a distinct pI value. Kumar et al. (2014) saw that the water solubility of a protein concentrate from *K. alvarezii* between pH 2 and 12 was ranked as follows: pure water > 0.1 M NaCl > 0.5 M NaCl.

### Extraction of algal proteins using the reference/basic protein extraction sequence

Using the described basic extraction sequence, a protein solubility of 34 % was achieved during the water incubation (i.e. the osmotic shock). Measuring the concentration of solubilized proteins after the second, alkaline extraction of the residual pellet from the first step, revealed a concentration of solubilized protein that was 29 % of that in the initial homogenate. Subtracting the volume loss from the removal of the pellets, the final total protein yields of the two steps were 25 and 20 %, respectively. The difference between protein solubilities in the two steps, 34 and 29 %, and total protein yields in the same steps reflect the retaining of protein-containing liquid in the pellet. The size of this loss could be dependent on several factors, such as water-holding capacity and centrifugation parameters.

### Effect of varying the length osmotic shock on the extraction yield

**Fig. 4:**
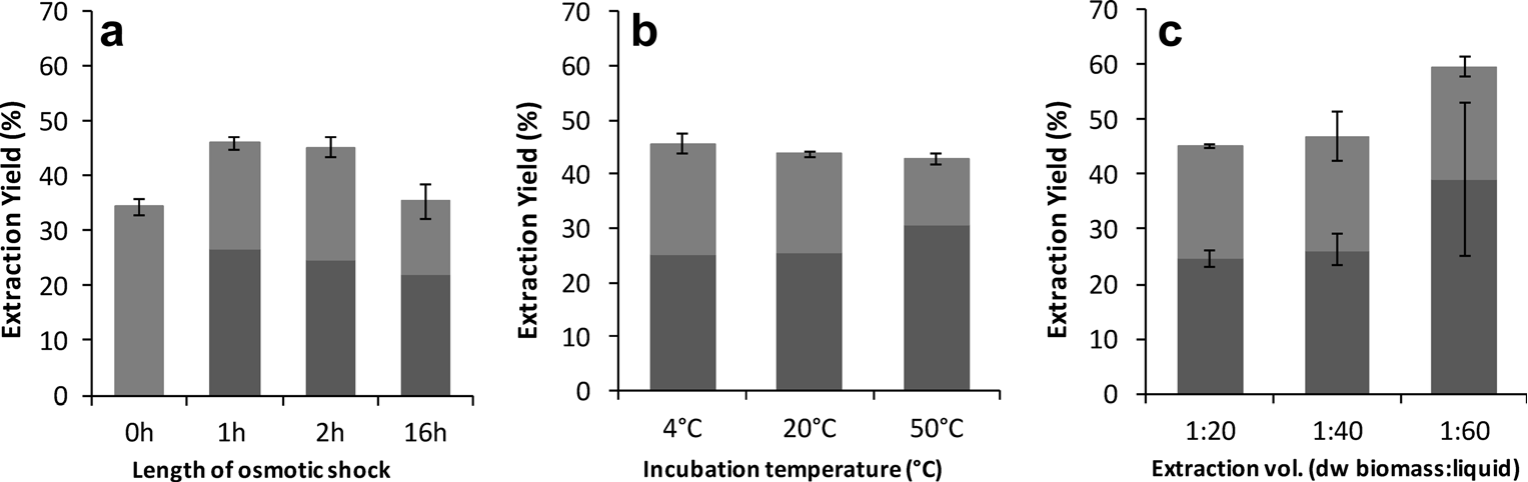
Effect on protein extraction yield of (a) length of osmoshock (using 1:20 dry biomass to water and incubation at 4 °C), (b) extraction volume (using 2-h incubation at 4 °C) and (c) temperature (using 1:20 dry biomass to water and 2-h incubation) on the efficiency of protein extraction. The *dark grey bars* represent the protein extracted in the separated water fraction (osmoshock), and the *light grey bars* represent the protein extracted in the alkaline fraction at pH 12. In graph a, the 0-h staple represents an experiment with no osmoshock but direct homogenization in alkaline conditions. All experiments were made in triplicates, and the *error bars* represent standard deviation

The osmotic shock was carried out to break cells, to facilitate the liberation of the algal proteins. To investigate the effect of the length of the osmotic shock on the amount of proteins solubilized by water and subsequently alkali, the length of the initial water incubation was varied from 0 to 16 h as shown in Fig. 4a. It appeared that the water incubation had a clear positive effect on the extractability but that it did not need to be long; there was no difference between 1- and 2-h incubations. These two incubation times gave total protein yields of 46 and 45 % after the subsequent alkaline extraction. There was a significant decrease in total extractability after the longest incubation that lasted 16 h, albeit the relative protein extractability in water was not significantly different from 1 and 2 h of osmotic shock. In the reference sample (0 h), which was not subjected to an osmotic shock prior to alkaline extraction, only 34 % of the total protein could be extracted.

### Effect of volume on extraction yield

The ratio between biomass and extraction liquid is known to be a factor that can affect the extraction yield. This is because more water allows for better solubility (Stefansson and Hultin 1994) and also creates a more diluted system, which causes less solubilized proteins to be retained in the pellet (Vareltzis and Undeland 2012). A higher proportion of water will also lower the ionic strength of the system, which could either increase or decrease protein solubility, depending on the shape of the salting-in/salting-out curve. In our study, we saw a positive effect on the extraction yield when the relative amount of water was increased from 1:20 to 1:60 (dw basis) (Fig. 4b). The effect was not pronounced, but still significant (*p* < 0.05). Just comparing the means, there was also an increase in extraction yield when the water ratio was increased from 1:40 to 1:60; this could, however, not be confirmed statistically, probably because of high variation between the replicates. When increasing the extraction volume to 1:60, especially the water extraction (i.e. the osmotic shock step) was positively affected with a 58 % increase in yield, compared to the reference extraction with 20 parts of water. The alkaline extraction, in fact, gave a somewhat lower yield at a 1:60 ratio, but the overall extraction yield was 59.5 %, which exceeds the yield obtained with the basic extraction protocol by 14.4 %.

### Effect of temperature on solubility

The temperature can be important for protein solubilization, and elevated temperatures can decrease solubility due to protein denaturation (González-Quijada et al. 2003). Denaturation is known to take place at lower temperatures, e.g. in fish compared to in warm-blooded animals (Howell et al. 1991). Our hypothesis was that algae, adapted to cold temperatures, would be less extractable at 20 or 50 °C compared to at 4 °C. However, in our experiments (Fig. 4c), there was no significant effect when varying the temperature between 4, 20 and 50 °C. Only in the initial water extraction, there was a slightly higher solubility at 50 °C, but this was balanced by a less effective alkaline extraction at this temperature, so that the final extraction yield was similar to that of 4 and 20 °C. The findings that extraction temperature had very little effect on the yield could be seen as a promising result, since it points towards lower requirements for energy during industrial extraction.

Based on the previous results, we suggest an extraction process at room temperature, with an algae/water ratio of 1:20 and a short water incubation of around 1 h for osmotic shock. If fresh water is not a limiting factor, a higher yield is indeed given with 60 volumes of water. Expressing the protein yields obtained with 20 vs 60 parts of water per 100 g ingoing dry algal biomass gives 3.98 and 5.25 mg g^−1^, numbers that can be compared with those of Harnedy and Fitzgerald (2013) working with *P. palmata*. They obtained a mean alkaline-soluble protein recovery of 5.76, 6.18 and 8.39 g (100 g)^−1^ dry algae weight, with osmotic shock, high shear treatments or addition of polysaccaridase enzymes (e.g. Celluclast 1.5 L and Shearzyme 500 L), respectively, to break down the cellular structures. Considering that the red seaweed *P. palmata* is known to be a more protein-rich species than brown seaweeds like *Saccharina latissima* (Fleurence 1999), the two-step extraction procedure in our study can be considered reasonably efficient.

### Precipitation at different pH values

**Fig. 5:**
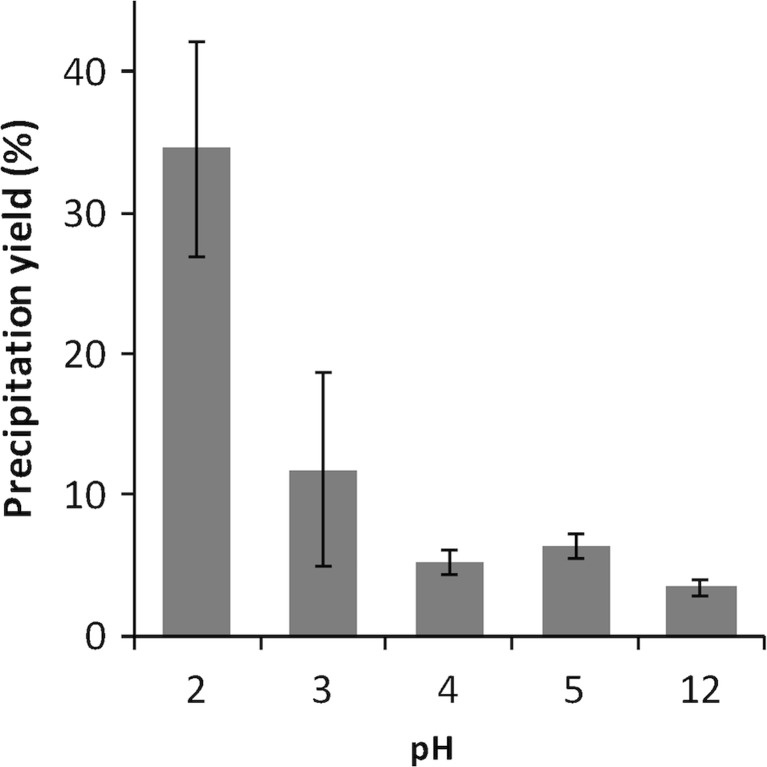
Efficiency of precipitation at different pH values. Aliquots of a protein extract of 0.81 mg mL^−1^ algal protein were subjected to a lowering of the pH to 5 through 2, and the precipitated protein fraction was separated by centrifugation. Yields are expressed as the percentage of the total protein content, in the original extract, that was retained in the pellets after separation. The original extract with a pH value of 12 was used as a control. All experiments were performed in triplicates, and the *error bars* represent standard deviations

To investigate a route to concentration of algal proteins based on alkali-aided solubilization, followed by isoelectric precipitation, the effect of pH on protein solubility in the acid range was investigated (Fig. 5). To better approach a putative industrial procedure, the extraction sequence was adjusted so that the water fraction was not separated after the osmotic shock but adjusted directly to pH 12, for a sequential alkaline extraction. After a centrifugation step, the alkaline extract was divided into aliquots for the precipitation experiment, with adjustment of pH to values from 2 to 5. At pH 4 and above, there was no protein precipitation as compared to the control (pH 12), whereas at pH 3 and pH 2, 11.7 and 34.5 % of the solubilized proteins were precipitated, respectively (Fig. 5).

The extraction yield of the water/alkali solubilization procedure (1:20, 2-h osmoshock, 20 °C) was 46.4 %, meaning that 33.6 mg of the ingoing 72.4 mg protein of the algal biomass could be released by extraction. In the following precipitation, pH 2 gave the highest yield, with precipitation of 34.5 % of the solubilized proteins. The overall isolation yield from our pH shift procedure was thus 16.01 %. In the work by Kandasamy et al. (2012), in which proteins were dissolved at pH 12 with 2-mercaptoethanol and precipitated with ammonium sulphate, ‘the percentage of recovered protein concentrate’ in three species of green seaweed was from 5.71 to 6.48 %; it is, however, not clear if this is a per weight unit or per total initial protein. The concentrates contained from 33.4 to 60.4 % protein (dw basis). Kumar et al. (2014), using the same protocol, reported on 7.81 % recovery of protein concentrate from *K. alvarezii* and the concentrate contained 62.3 % protein. Wong and Cheung (2001), also applying the same protocol, reported 7.8–48 % protein recovery from dried *Sargassum* species. They found a negative correlation (*r* = −0.96) between protein extractability and the total phenolic compound level in the range 10.5 to 24.5 mg phenolics g^−1^ dw of the three species dried with two different drying methods (freeze drying, oven drying). Phenolics may form reversible hydrogen bonds with proteins or oxidize to quinones, which irreversibly can bind to proteins, limiting protein extractability*. Saccharina latissima* harvested on the Swedish west coast in the late fall, as in this study, has previously been reported to contain 8.2 mg phenolic compounds g^−1^ dw (Veide Vilg et al. 2015), and such molecules could, indeed, be a factor that negatively affects the extractability, also in this study. With regard to all the listed studies, it should be stressed that ammonium sulphate-driven precipitation, however, reinforces the need for an extra dialysis step that complicates the process.

Calculating with a dry weight protein content of 9 %, as in our biomass, and a production yield of 130 t of cultivated kelp biomass per hectare (Kraan 2013), 18 % dry matter and a 16 % extraction yield would give around 340 kg protein per hectare of cultivation. To reach the level of productivity from soy cultivation, which is around 400 kg (edible) soy protein per hectare (Kaldy 1972), an extraction yield of at least 19 % needs to be achieved. There are several factors that could be investigated to improve the yield. In our experiments, we used relatively low g-forces during centrifugation; applying a more forceful centrifugation could give a more efficient separation of the liquid from the solid phase, with less retention of solubilized protein in the pellet as an effect. We can also hypothesize that the use of carbohydrate-degrading enzymes, specifically alginate lyases, would lower the water-holding capacity of the pellet, due to the degradation of the highly gelling kelp compound alginate. Another possible effect of addition of carbohydrate-degrading enzymes could be that their degradation of structural components of the cell wall would increase the release of proteins into solution. It was previously shown that a very high enzyme concentration was needed to achieve any effect on protein yield (Harnedy and FitzGerald (2013), but since the enzyme formulas used in that report were designed for degradation of terrestrial plant material, the general effect of enzymatic treatment could be underestimated. To clarify the feasibility of enzymatic degradation of algal biomass for protein extraction, the enzyme mixtures would need to be tailor-made for the composition of the cell walls of the specific algal species. The precipitation yield could also possibly be improved by adding, e.g. flocculating/precipitating agents, provided they are food grade and thereby acceptable in the final product. As for the separation following extraction, the g-forces of centrifugation could be increased also for the precipitation step, to improve the overall yield. This would, however, increase the energy costs. Other possible adjustments could be to lengthen the precipitation incubation period and to lower the temperature. Further investigations are thus needed to develop an optimal protein concentration protocol for *Saccharina latissima*, but this study can be seen as an initial mapping of factors influencing the procedure.
